# Physics informed contour selection for rapid image segmentation

**DOI:** 10.1038/s41598-024-57281-x

**Published:** 2024-03-24

**Authors:** Vikas Dwivedi, Balaji Srinivasan, Ganapathy Krishnamurthi

**Affiliations:** 1 Atmospheric Science Research Center, State University of New York, Albany, NY 12222 USA; 2 Department of Mechanical Engineering, Indian Institute of Technology, Madras, Chennai, 600036 India; 3 Department of Engineering Design, Indian Institute of Technology, Madras, Chennai, 600036 India; 4 Wadhwani School of Data Science and AI, Indian Institute of Technology, Madras, Chennai, 600036 India

**Keywords:** Applied mathematics, Computational science, Applied mathematics, Computational science

## Abstract

Effective training of deep image segmentation models is challenging due to the need for abundant, high-quality annotations. To facilitate image annotation, we introduce Physics Informed Contour Selection (PICS)—an interpretable, physics-informed algorithm for rapid image segmentation without relying on labeled data. PICS draws inspiration from physics-informed neural networks (PINNs) and an active contour model called snake. It is fast and computationally lightweight because it employs cubic splines instead of a deep neural network as a basis function. Its training parameters are physically interpretable because they directly represent control knots of the segmentation curve. Traditional snakes involve minimization of the edge-based loss functionals by deriving the Euler–Lagrange equation followed by its numerical solution. However, PICS directly minimizes the loss functional, bypassing the Euler Lagrange equations. It is the first snake variant to minimize a region-based loss function instead of traditional edge-based loss functions. PICS uniquely models the three-dimensional (3D) segmentation process with an unsteady partial differential equation (PDE), which allows accelerated segmentation via transfer learning. To demonstrate its effectiveness, we apply PICS for 3D segmentation of the left ventricle on a publicly available cardiac dataset. We also demonstrate PICS’s capacity to encode the prior shape information as a loss term by proposing a new convexity-preserving loss term for left ventricle. Overall, PICS presents several novelties in network architecture, transfer learning, and physics-inspired losses for image segmentation, thereby showing promising outcomes and potential for further refinement.

## Introduction

Image segmentation^1^ involves identifying and delineating specific regions or objects within an image. The approaches to image segmentation can be broadly categorized into two extremes: deep learning-based models^2^ that rely on substantial labeled training data and, traditional active contour models^3^ that do not require training data, but face challenges related to some theoretical and numerical aspects. Deep learning-based segmentation models have been proven to be highly successful^4–6^ but their effectiveness is constrained by the requirement for abundant labeled data, posing limitations in situations where data is scarce or unavailable^7–9^. To tackle the unlabeled data issue, the first question that we ask is whether active contour models can be employed to produce high-quality annotations for training deep learning models.

Among all active contour models, snake^10^ is the most intuitive image segmentation model. It is based on the concept of a deformable curve or surface that can be iteratively adjusted to fit the edges or boundaries of an object in an image by solving a system of PDEs known as Euler–Lagrange equations. Despite being very intuitive, snake models are mathematically complex, and suffer from various issues like sensitivity towards initialization and data noise, difficulty in using prior-shape information, etc. In this work, we propose that many of these limitations can be addressed by combining traditional snakes with PINNs^11,12^, a relatively new machine learning approach for solving PDEs. A concise review of PINN and snake model is available in the supplementary notes.

This paper develops a new label-free image segmentation algorithm called PICS (Physics Informed Contour Selection), which combines snakes and PINNs by introducing several novelties to the original PINN approach. To demonstrate the effectiveness of PICS, we take an example from the field of medical image segmentation. We apply PICS for 3D segmentation of the left ventricle on a publicly available cardiac dataset^13^. We also demonstrate PICS’s capacity to encode the prior shape information as a loss term by proposing a new convexity-preserving^14^ loss term for left ventricle.

The paper is structured as follows: In the “Methods” section, we present the mathematical formulation of PICS, highlighting its similarities and differences with PINN, and elucidating how it addresses the challenges associated with the snake model. Subsequently, in the “Results” section, we analyze the performance of PICS in 2D and 3D segmentation across diverse test cases. Finally, we delve into the limitations of PICS and provide concluding remarks in the “Discussion” section.

## Methods

**Figure 1:**
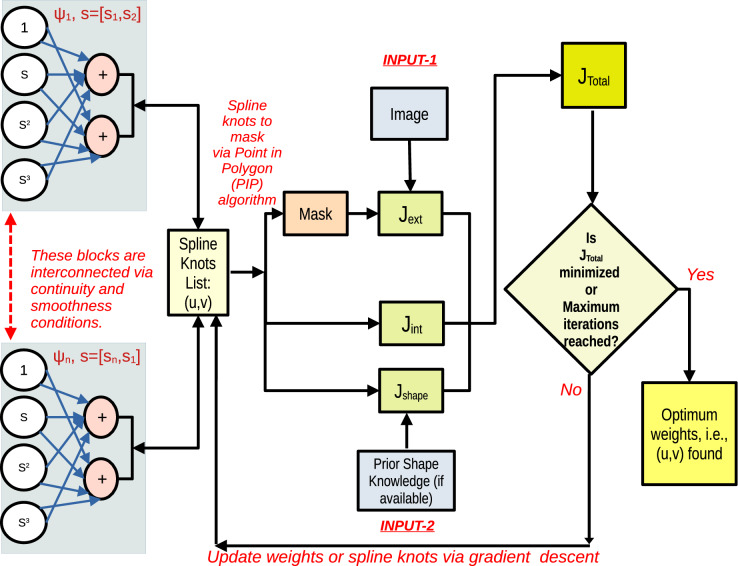
The algorithm takes as input an image (INPUT-1) and, optionally, prior shape knowledge (INPUT-2) of the target in certain cases. The user initiates the snake by hovering over the image and clicking on it once. The initial weights of the snake denote the control knots of the cubic spline, functioning as PICS’s hypothesis. Subsequently, we compute losses based on the internal and external energy of the snake model, and optionally, a prior-shape-based loss. The total loss is minimized through gradient descent, leading to updates in the values of spline control knots.

PICS aims to achieve segmentation for a given 2D or 3D image. In scenarios where experts possess information about the shape of the target object, PICS leverages this information to enhance its performance. The flowchart of the PICS algorithm is depicted in Fig. 1. In this section, we will go into details of its individual components, i.e., (a) PICS hypothesis, (b) the loss function and optimization,(c) the prior shape-based loss term, and (d) the operation performance index (OPI)–a metric to monitor the optimization performance of PICS.

### PICS hypothesis

We approximate the target solution, i.e., object boundary with a parametric spline. The expression of parametric spline $\vec {\psi }$ is given by

1 $$\begin{aligned} \vec {\psi }(s)={\left\{ \begin{array}{ll} \begin{array}{c} \vec {\psi }_{1}\\ \vec {\psi }_{2}\\ \ldots \\ \vec {\psi }_{n} \end{array} &{}\quad \begin{array}{c} s_{1}<s<s_{2}\\ s_{2}<s<s_{3}\\ \ldots \\ s_{n}<s<s_{1} \end{array}\end{array}\right. } \end{aligned}$$

where the local cubic spline $\vec {\psi }_{i}(s_{i}<s<s_{i+1})$ is given by

2 $$\begin{aligned} \vec {\psi _{i}}(s)=\left\{ \begin{array}{c} u_{i}(s)\\ v_{i}(s) \end{array}\right\} =\left\{ \begin{array}{c} a_{i}s^{3}+b_{i}s^{2}+c_{i}s+d_{i}\\ e_{i}s^{3}+f_{i}s^{2}+g_{i}s+h_{i} \end{array}\right\} \end{aligned}$$

In the given equation, *s* is a parameter that varies from 0 to 1. $[u_{i},v_{i}]'$ denotes the spatial coordinates of the local spline $\psi _{i}$. For given $[u_{i},v_{i}]'$ , the coefficients of local splines, denoted by $[a_{i},b_{i},\ldots ,h_{i}]'$, are computed by satisfying the conditions of continuity, smoothness, and periodicity. Mathematically,

1. Continuity: $$\vec {\psi }_{k-1}(s_{k})=\vec {\psi }_{k}(s_{k})=\left\{ \begin{array}{c} {{u}}(s_{k})\\ {{v}}(s_{k}) \end{array}\right\}$$
2. Smoothness: $$\frac{d}{ds}\vec {\psi }_{k-1}(s_{k})=\frac{d}{ds}\vec {\psi }_{k}(s_{k})$$ and $$\frac{d^{2}}{ds^{2}}\vec {\psi }_{k-1}(s_{k})=\frac{d^{2}}{ds^{2}}\vec {\psi }_{k}(s_{k})$$
3. Periodicity: $$\frac{d}{ds}\vec {\psi }_{n}(s_{n})=\frac{d}{ds}\vec {\psi }_{1}(s_{1})$$ and $$\frac{d^{2}}{ds^{2}}\vec {\psi }_{n}(s_{n})=\frac{d^{2}}{ds^{2}}\vec {\psi }_{1}(s_{1})$$

#### Remarks

Conceptually, PICS architecture can be viewed as a special case of Distributed PINNs^12^, where localized shallow PINNs are employed. In the later sections, we will note that 3D segmentation is also inspired by time-marching Distributed PINNs. However, there are distinctions between PINN and PICS which are outlined as follows:

- *Architecture*: PINNs use a deep neural network with a large number of parameters to approximate the solution, whereas PICS employs cubic splines that can efficiently approximate any closed contour with only a few control knots. It is already reported by Dwivedi and Srinivasan^15^ that the use of simplified architectures improves the speed of PINN.
- *Mathematical nature*: PINN’s hypothesis is nonlinear. In contrast, the hypothesis in PICS is linear.
- *Interpretation of weights*: Unlike in PINN, the spline coefficients are not directly considered trainable weights in PICS. In PICS, the weights are the sampling points or the control knots themselves, i.e., $[u_{i},v_{i}]'$. There are two main reasons for preferring control knots over spline coefficients:
  1. Opting to directly choose spline coefficients results in a large number of weights, making it impossible for us to visualize and physically interpret loss gradients in a 2D plane. Conversely, if we designate the control knots as weights, any number of weights can be represented in a 2D plane, and the loss gradient then physically signifies the force acting on the control knots.
  2. Weight decay is often used for good generalization during the training of deep neural networks. However, weight decay is purely a data-driven technique. In contrast, PICS weights are subject to physical constraints such as continuity, smoothness, and periodicity. Consequently, any erratic oscillation in one weight is counteracted by the collective influence of all the others.

### Loss function and optimization

PICS combines snake’s internal energy terms^10^ with a Chan–Vese loss^16,17^ term for external energy. Chan–Vese loss functional is less sensitive to noisy pixel-level annotations, which is beneficial when dealing with imperfect or noisy ground truth data. The formula for the loss function is given by

3 $$\begin{aligned} J=\alpha J_{\psi _{s}}+\beta J_{\psi _{ss}}+\mu J_{cv} \end{aligned}$$

where

4 $$\begin{aligned} J_{\psi _{s}}= & {} \sum _{i=1}^{i=N}\frac{1}{N}\left( \left( \frac{d{\widetilde{u}}}{ds} \right) ^{2}+\left( \frac{d{\widetilde{v}}}{ds}\right) ^{2}\right) _{i} \end{aligned}$$

5 $$\begin{aligned} J_{\psi _{ss}}= & {} \sum _{i=1}^{i=N}\frac{1}{N}\left( \left( \frac{d^{2} {\widetilde{u}}}{ds^{2}}\right) ^{2}+\left( \frac{d^{2}{\widetilde{v}}}{ds^{2}}\right) ^{2}\right) _{i} \end{aligned}$$

6 $$\begin{aligned} J_{cv}= & {} \sum _{q=1}^{q=N_{y}}\sum _{p=1}^{p=N_{x}}\left( \left( I(p,q) -\mu _{in}\right) \chi (p,q)\right) ^{2}+\gamma \left( \nabla I(p,q) \chi (p,q)\right) ^{2}+\left( \left( I(p,q)-\mu _{out}\right) (1-\chi (p,q))\right) ^{2} \end{aligned}$$

In the above expressions, *I* denotes the image, $({\widetilde{u}},{\widetilde{v}})$ denotes spline knots, and *N* denotes the number of spline control knots. $\chi$ denotes a characteristic function or mask that is generated by repeated geometric queries, that is, given a single polygon through spline knots and a sequence of query points (grid points), find if the query point lies inside or outside the polygon using point in polygon algorithms^18^. $\mu _{in},\mu _{out}$ denote the average pixel value of the image within and outside the spline contour. $N_{x},N_{y}$ denote number of pixels in *x* and *y* direction respectively. With respect to Fig. 1, the external energy term of the loss is $J_{ext}=\mu J_{cv}$, and the internal energy term is $J_{int}=\alpha J_{\psi _{s}}+\beta J_{\psi _{ss}}$. The hyperparameter $\gamma$ aims to make the pixel intensities inside the contour more uniform.

While doing the weight update by gradient descent, the derivative of loss with respect to weights is compulsory. For example, The expression for weight update using gradient descent is given by

7 $$\begin{aligned} w=w_{old}-\lambda \frac{\partial J}{\partial w_{old}} \end{aligned}$$

where *w* denotes weight or spline control knots, $\lambda$ is the learning rate and $\frac{\partial J}{\partial w}$ is the loss gradient.

#### Remarks

- The loss gradient cannot be calculated directly or even by automatic differentiation^19^ because there is no explicit differentiable function that maps control knots with mask. Therefore to calculate derivatives, we use central difference scheme. This is the advantage of minimizing the energy functional instead of using PINN-like PDE residual, as it relaxes the differentiability requirements. For faster convergence and adaptive learning rate, we have used Adam^20^ optimizer for our numerical experiments.
- In most deep neural networks, the weights are typically initialized randomly and do not have any physical significance. However, PICS is formulated in such a way that trainable weights are represented by the control knots of cubic splines. It gives it a clear physical meaning to weights that simplifies scaling and normalization steps. Similarly, the loss gradient in PICS can be physically interpreted as the *force* on the control knots pushing them towards the direction of gradient descent.

### Prior shape-based loss term

In cases where domain experts possess information about the shape of the target object, PICS can leverage this valuable information. This is accomplished by introducing prior-shape based loss terms, allowing the algorithm to incorporate and benefit from the expert-provided shape information. This paper will use PICS to generate annotations for the left ventricle in the cardiac MRI scan images.

**Figure 2:**
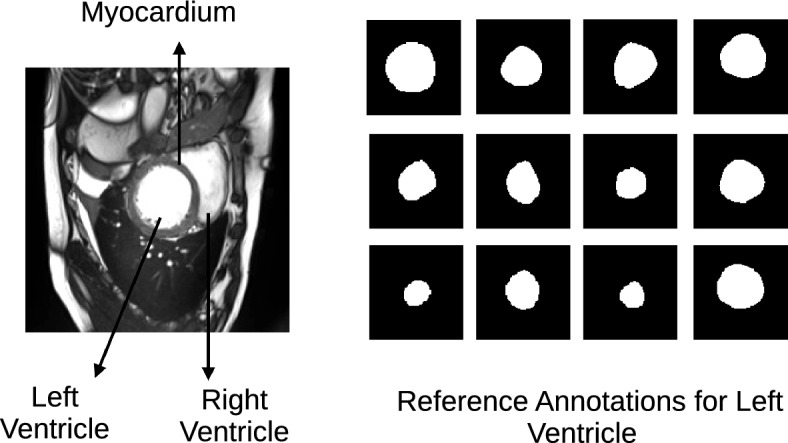
Description of the cardiac dataset and sample annotations for the left ventricle.

- *Domain knowledge*: A representative cardiac MRI scan shown in the left-hand side of Fig. 2 is composed of three main parts: left ventricle, right ventricle, and myocardium. In clinical applications of cardiac left ventricle (LV) segmentation, it is desirable to include the cavity, trabeculae, and papillary muscles, which collectively form a convex shape, as shown by the right-hand side of Fig. 2 where some reference annotations for left ventricle are depicted.
- *Why left ventricle?*: Trabeculae and papillary muscles have similar intensities to the myocardium, which can cause segmentation algorithms to incorrectly classify them as myocardium. The problem here is to find a way to accommodate medical domain knowledge with a purely data or image-driven algorithm.
- *Proposed loss term*: To address this challenge, Shi and Li^14^ developed a method that preserves the convexity of the left ventricle by controlling the curvature in the level set framework. Similarly, in PICS, we propose a new loss term that preserves convexity in the snake framework. This loss term is expressed as follows:
  8 $$\begin{aligned} J_{shape}=\sigma \sum _{i=1}^{i=N}\frac{1}{N}\kappa ^{2}= \sigma \sum _{i=1}^{i=N}\frac{1}{N}\left( \frac{{\tilde{v}}_{ss} {\tilde{u}}_{s}-{\tilde{u}}_{ss}{\tilde{v}}_{s}}{({\tilde{u}}_{s}^{2}+ {\tilde{v}}_{s}^{2})^{\frac{3}{2}}}\right) ^{2} \end{aligned}$$
  where $\kappa$ denotes the curvature of contour, *N* denotes number of spline control knots and $\sigma$ is a hyperparameter. This penalty term ensures that the shape of the predicted boundary remains convex-shaped. Please note that such an information about the shape of the object is not always available. In those cases, as Fig. 1 shows, PICS works with just $J_{int}$ and $J_{ext}$.

### Operation performance index (OPI)

The region-based loss function is comprised of both shape regularization and external energy (or mean square error) terms. During optimization, our aim is to reduce the total loss. However, if the relative strengths of the shape regularization and MSE terms need to be appropriately balanced, the solution may get stuck in a local minimum.

To prevent this issue, we introduce a new performance evaluation metric known as the Operation Performance Index (OPI). A value of one for OPI indicates that PICS is moving in the right direction, while a value below a predetermined threshold (e.g., 0.8) indicates that parameter adjustments are necessary. However, what constitutes the “right direction”? In the best-case scenario, both external and internal energies should drop as optimization proceeds. However, if that is not possible, the external energy should always drop whether shape regularization loss drops or not. This idea is mathematically contained in the following formula for OPI:

9 $$\begin{aligned} OPI_{k}=1-\frac{\langle \vec {\theta _{k}},\vec {P_{k}}\rangle }{2\sum \vec {\theta _{k}}} \end{aligned}$$

where

$$\begin{aligned} \vec {P_{k}}= & {} sign(\triangle J_{int}(k-w+1:k))-sign(\triangle J_{ext}(k-w+1:k))\\ \vec {\theta _{k}}= & {} \frac{exp([1,1+d, \ldots 1+id, \ldots 2])}{\sum _{i=0}^{i=w-1}exp(1+id)},d=\frac{1}{w-1} \end{aligned}$$

*k* , *w*, sign, and $\langle \rangle$ represent iteration number, iteration window size, signum function, and dot product, respectively. $\triangle J$ represents vector of difference in *J*. The exponential smoothing term ensures that the recent values are given more weightage.

OPI can also be used for hyperparameter tuning. For instance, the values of the hyperparameters $\alpha$ and $\beta$ can be determined through trial and error. But, the third parameter, $\mu$, which is initially set to 1e3, can be adjusted using OPI. When OPI falls below the threshold, the update rule for $\mu$ is given by

10 $$\begin{aligned} \mu =\mu +2^{log_{10}\left( J_{ext}/J_{int}\right) } \end{aligned}$$

However, we do not continue adjusting $\mu$ indefinitely. We stop adjusting $\mu$ once the order of $J_{ext}$ becomes more than 1e4 times that of $J_{int}$ to prevent the snake from becoming too loose.

When examining the total loss history alone, it is difficult to determine whether the optimization is progressing correctly. However, looking at the OPI trend, we can check if its value is very low or wildly fluctuating between 0 and 1. Based on this information, the hyperparameters may be adjusted. We will cover the application of this idea in the next section.

### Rapid 3D segmentation with transfer learning

If *F* represents a nonlinear transformation that takes image *I*(*x*, *y*) and initial weights $\vec {w}$ as input and gives weight update as output. We can model the 3D segmentation process as follows:

11 $$\begin{aligned} \vec {w}^{[n]}=\vec {w}_{opt}^{[n-1]}+F(I(x,y)^{[n]},\vec {w}_{opt}^{[n-1]}),\quad n=1,2,3,.. \end{aligned}$$

where the weights are initialized by a single mouse click of the user, i.e.,

12 $$\begin{aligned} \vec {w}^{[0]}=\vec {w}_{user} \end{aligned}$$

It is mathematically equivalent to solving an unsteady PDE given by

13 $$\begin{aligned} \frac{d\vec {w}}{dt}=F(I(x,y),\vec {w}) \end{aligned}$$

This PDE approach is inspired by Distributed PINNs^12^, and the effect of transfer learning is “spatially-aware initialization” from second slice onwards.

#### Remarks

This section described the PICS methodology and provided a comparative analysis with PINN. Before proceeding to the next section, it’s important to emphasize how this methodology effectively addresses certain limitations inherent in traditional snakes.

- *Sensitivity to initialization*: One of the primary challenges with snakes is their sensitivity to initialization. If the contour is initialized far from the actual boundaries, it might not converge due to the presence of local minima. In the case of PICS, a domain expert clicks on the image, ensuring that the initial snake is within the image and not too distant from the true boundary.
- *Sensitivity to noise*: Chan–Vese loss functional is less sensitive to noisy pixel-level annotations, which is beneficial when dealing with imperfect or noisy ground truth data.
- *Complicated mathematical formulation*: PICS directly works with loss functionals, eliminating the necessity of deriving Euler–Lagrange equations. The optimization process involves straightforward gradient descent, facilitating the minimization of all types of loss functionals.
- *Difficult to incorporate shape-priors*: PICS can easily incorporate shape priors as loss terms.

## Results

In this section, we demonstrate the effectiveness of PICS in 2D and 3D segmentation with and without prior shape information. In all the cases, Adam optimizer^20^ is used. All the experiments are conducted in Matlab R2022b environment running in a 12th Gen Intel(R) Core(TM) i7-12700H, 2.30 GHz CPU and 16GB RAM Asus laptop.

### Test cases

For testing PICS, we have considered the following cases:

- **2D test cases** : (TC-1) 2D segmentation of CT scan of enlarged ventricles of hydrocephalus patient (Case courtesy of Paul Simkin, Radiopaedia.org, rID: 30453). The source of data is: https://radiopaedia.org/cases/obstructive-hydrocephalus, (TC-2A, TC-2B) 2D segmentation (without and with shape prior loss) of MRI scan with indistinct left ventricle from ACDC dataset^13^.
- **OPI test cases**: (TC-3A, TC-3B, TC-3C, TC-4) 2D segmentation without and with adaptive hyperparameters of synthetic image of a cavity^21–23^. This is a standard test case where traditional snake models have been observed to be unsuccessful in navigating through concavities, (TC-4) 2D segmentation of Texas state from the map of the United States of America (USA).
- **3D test cases**: (TC-5) 3D segmentation of MRI scans of cardiac data of 100 patients from the ACDC dataset^13^ in the ED, i.e., End-Diastolic phase. Source:https://www.creatis.insa-lyon.fr/Challenge/acdc/index.html

### Evaluation metrics

- **Qualitative evaluation**: Except for the ACDC dataset images, the objects in both 2D and OPI test cases are simple and can be assessed visually without the need of any expert labels.
- **Quantitative evaluation**: The last test, which uses the ACDC dataset, requires expert interpretation and therefore is evaluated by comparing the results with annotations provided by medical professionals. The Dice score is used as the evaluation metric to compare the results. The formula for dice score is
  $$\begin{aligned} Dice(A, B) = 2\frac{|A \cap B|}{|A |+| B|} \end{aligned}$$
  where A and B are the two sets being compared. The Dice value ranges from 0 to 1, where 0 indicates no common elements between the sets and 1 indicates identical sets.

### Hyperparameter selection

The loss function in PICS comprises internal and external energy terms. The hyperparameters govern the relative significance of these terms, and their absolute values do not hold any particular significance. Consequently, there is no unique search space for hyperparameters. In this paper, the process of hyperparameter selection has not been automated and is definitely a topic of future work. Nevertheless, the adopted trial-and-error approach is as follows:

- Begin by focusing on internal energy terms. Choose some values for $(\alpha ,\beta )$ such that the orders of magnitude of $\alpha \psi _{s}^{2}$ and $\beta \psi _{ss}^{2}$ are comparable for simple shapes like circle and rectangle. For this, typically the order of magnitude of $\beta$ is one or two less than order of magnitude of $\alpha$. For example if $\alpha =0.1$, then $\beta$ = 0.01 or 0.001.
- Moving on to the external energy term, the value of $\mu$ is fine-tuned to ensure that the order of magnitude of the Chan–Vese loss is not lower than that of the internal energy term. In any case, the decrease in internal energy should not be favored over the increase in external energy. OPI-based hyperparameter tuning essentially accomplishes this. The coefficient of the image gradient, denoted as $\gamma$, is typically set to zero but may be adjusted only in special cases when the object boundary is significantly blurred.
- Lastly, when there is prior shape information available for the Left Ventricle, the value of $\sigma$ needs to be set. In typical scenarios, its value is kept low. However, for the indistinct muscles category, where domain expertise should take precedence over data, the value of $\sigma$ is increased by a factor of 10. This adjustment ensures that prior shape-based loss dominates over the Chan–Vese loss terms.

### Control knots initialization

The initial state of the snake is set as a circle, typically with a relatively small radius compared to the object intended for segmentation. The user determines the circle’s center position by hovering over the image and clicking the mouse. The control points are evenly spaced along the circle’s circumference.

### Performance

#### 2D cases (TC-1, TC-2)

**Figure 3:**
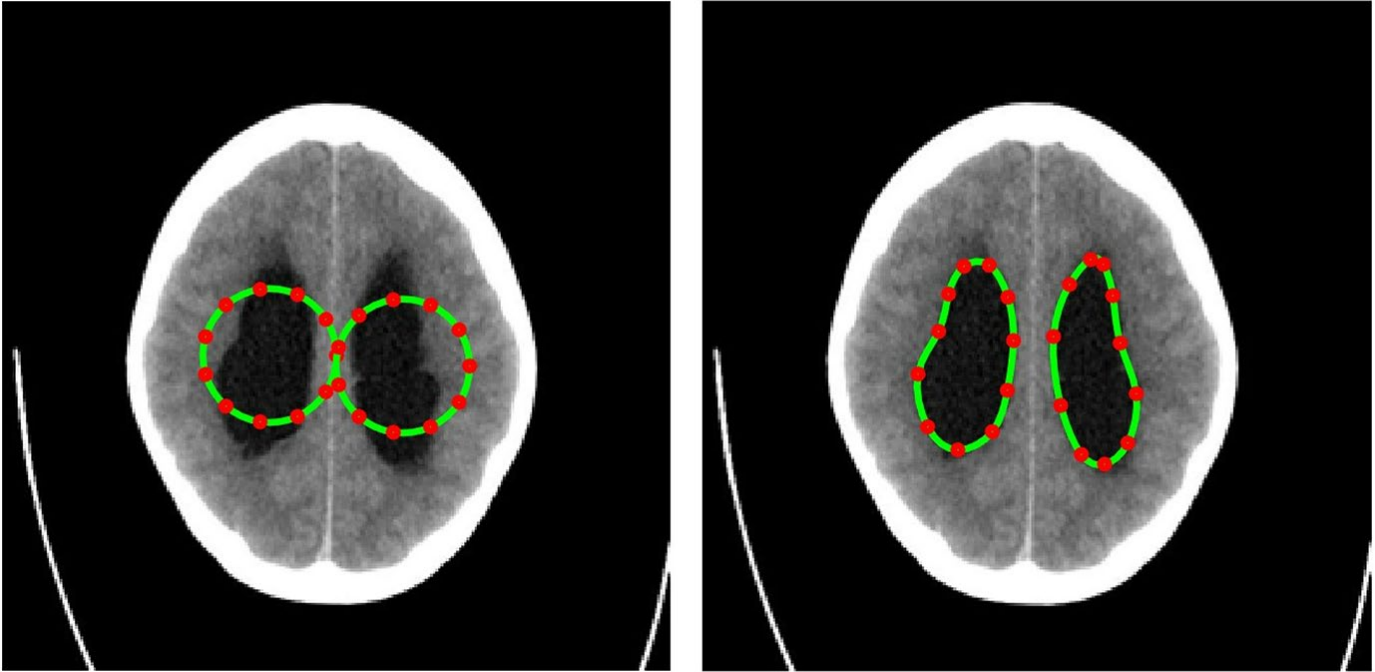
Segmentation of enlarged ventricles of a hydrocephalus patient. Left: Initial weights, Right: Optimized weights.

**Figure 4:**
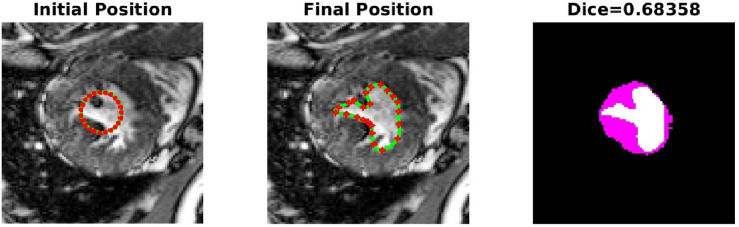
Low Dice score: Segmentation of the left ventricle without convexity preserving shape prior.

**Figure 5:**
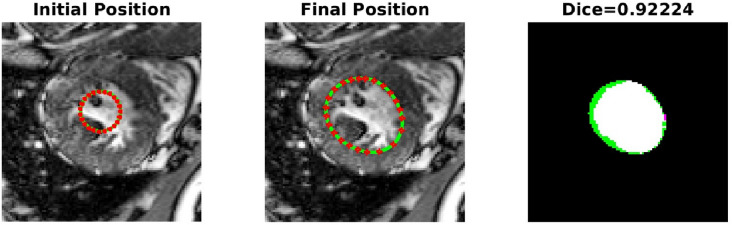
High Dice score: Effect of inclusion of convexity preserving shape prior in segmentation of the left ventricle.

**Figure 6:**
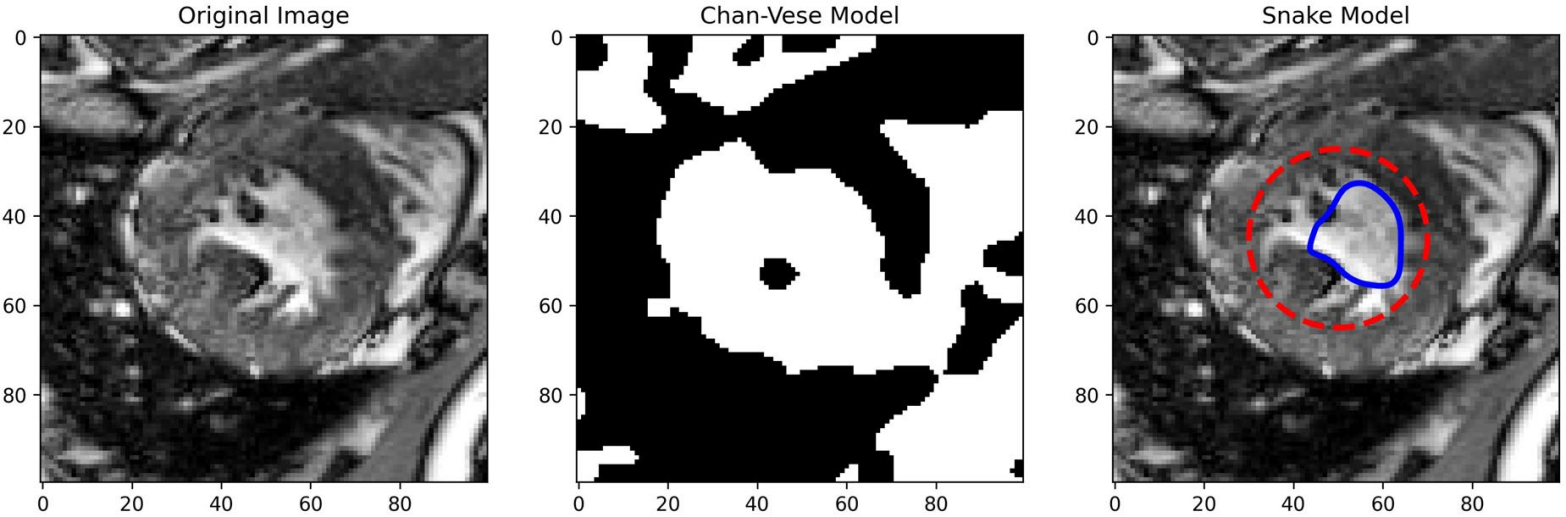
Segmentation with the traditional Chan–Vese and snake models.Chan–Vese model does not distinguish between the bright intensities of the left and right ventricle. Snake model sticks to the region with sharp gradients.

The PICS settings for all the 2D cases are summarized in Table 1. The main findings from the 2D cases are as follows:

- **TC-1**: In the first case, we consider a CT scan of the enlarged ventricles of a hydrocephalus patient. Figure 3 shows the segmentation results. The CT scan shows two enlarged ventricles. The left-hand side of Fig. 3 shows the contours initialized by the PICS user, and the right-hand side figure shows the optimized weights. By visual inspection, we can conclude that the results are satisfactory and PICS shows good performance when dealing with images that contain a single target object or when the number of target objects is known.
- **TC-2A,TC-2B**: In the second case, Figs. 4 and 5 demonstrate the effect of integrating a convexity-preserving loss term in the segmentation of the left ventricle. As depicted in Fig. 4, a purely data-driven segmentation algorithm fails in cases where trabeculae and papillary muscles have comparable intensities to the myocardium. However, by incorporating prior shape information that preserves convexity, PICS is able to accurately segment the left ventricle even in the presence of confusing muscles, as shown in Fig. 5. The inclusion of the shape loss term results in an increase of the Dice score from 0.68 without the shape loss term to 0.92 with it. We also show the segmentation output with traditional Chan–Vese (https://scikit-image.org/docs/stable/auto_examples/segmentation/plot_chan_vese.html) and snake (https://scikit-image.org/docs/stable/auto_examples/edges/plot_active_contours.html) models in Fig. 6. In the level set formulation, the Chan–Vese model does not distinguish between the bright intensities of the left and right ventricle. On the other hand, the traditional snake model, even when initialized very close to the true mask, adheres to locations with sharp pixel gradients.

**Table 1:** Experiment settings for 2D and OPI test cases. Columns 2 to 6 have values of loss coefficients. $N_{w}$ in column 7 denotes number of PICS weights. Last column has convergence times. TC-4 takes longer time than others because TC-4 image is of size $256 \times 256$, and all others are $128 \times 128$.

Test case	$\alpha$	$\beta$	$\mu$	$\gamma$	$\sigma$	$N_{w}$	Time (s)
TC-1	1e−1	1e−3	1e4	0	0	44	20
TC-2A	1e−1	1e−2	5e4	0	0	46	30
TC-2B	5e−1	1e−3	5e4	0	5e8	46	81
TC-3A	5	1e−1	1e2	0	0	46	40
TC-3B	5e−1	1e−2	1e2	0	0	46	76
TC-3C	5e−1	1e−2	1e3	0	0	70	332
TC-4	8e−1	1e−3	1e3	0	0	46	175

#### OPI cases (TC-3A, 3B, 3C and TC-4)

**Figure 7:**
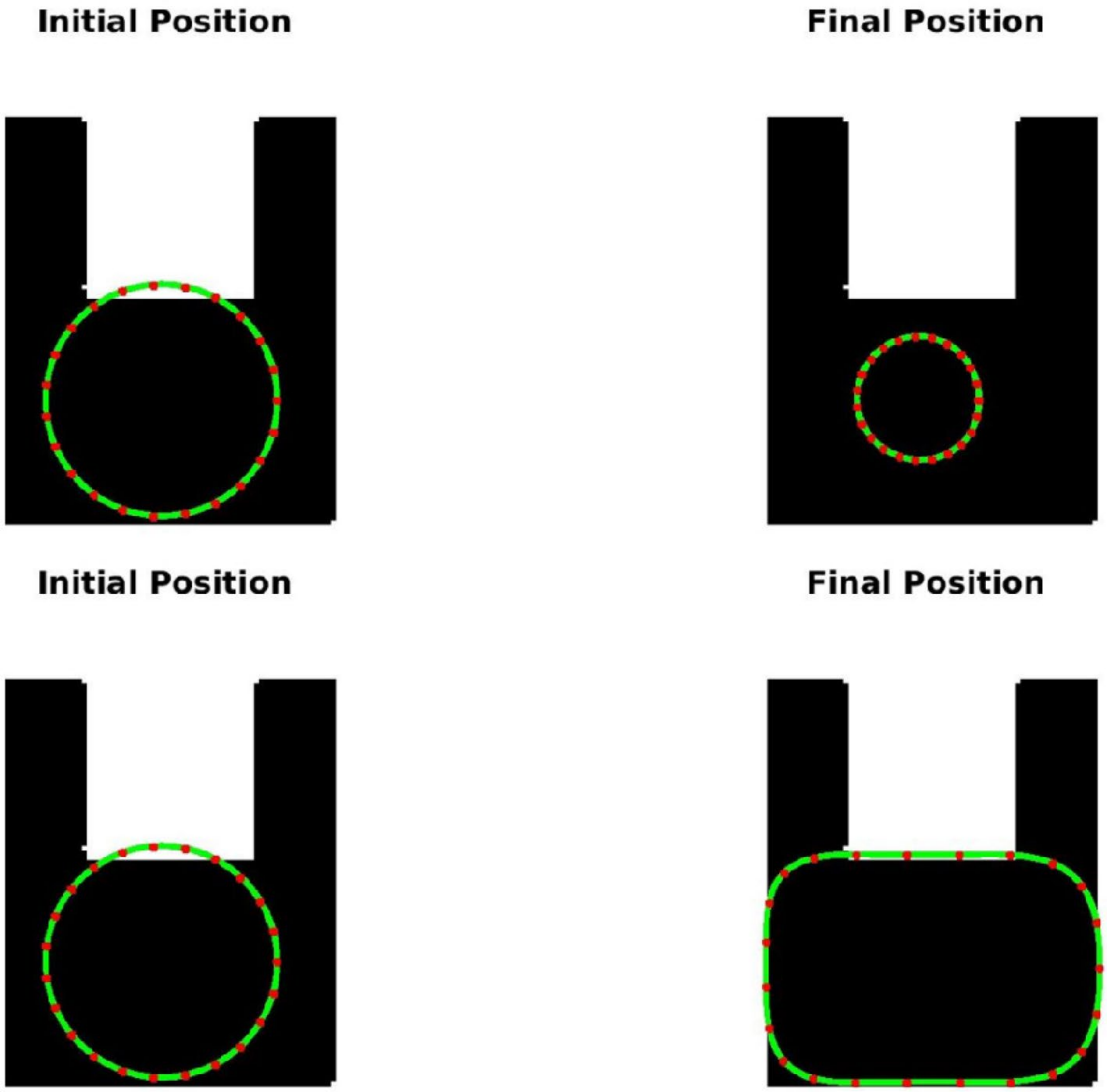
Two examples of bad minima due to loss function. In both the cases, the order of magnitude of drop in internal energy is higher (or comparable) than that of drop in external energy.

**Figure 8:**
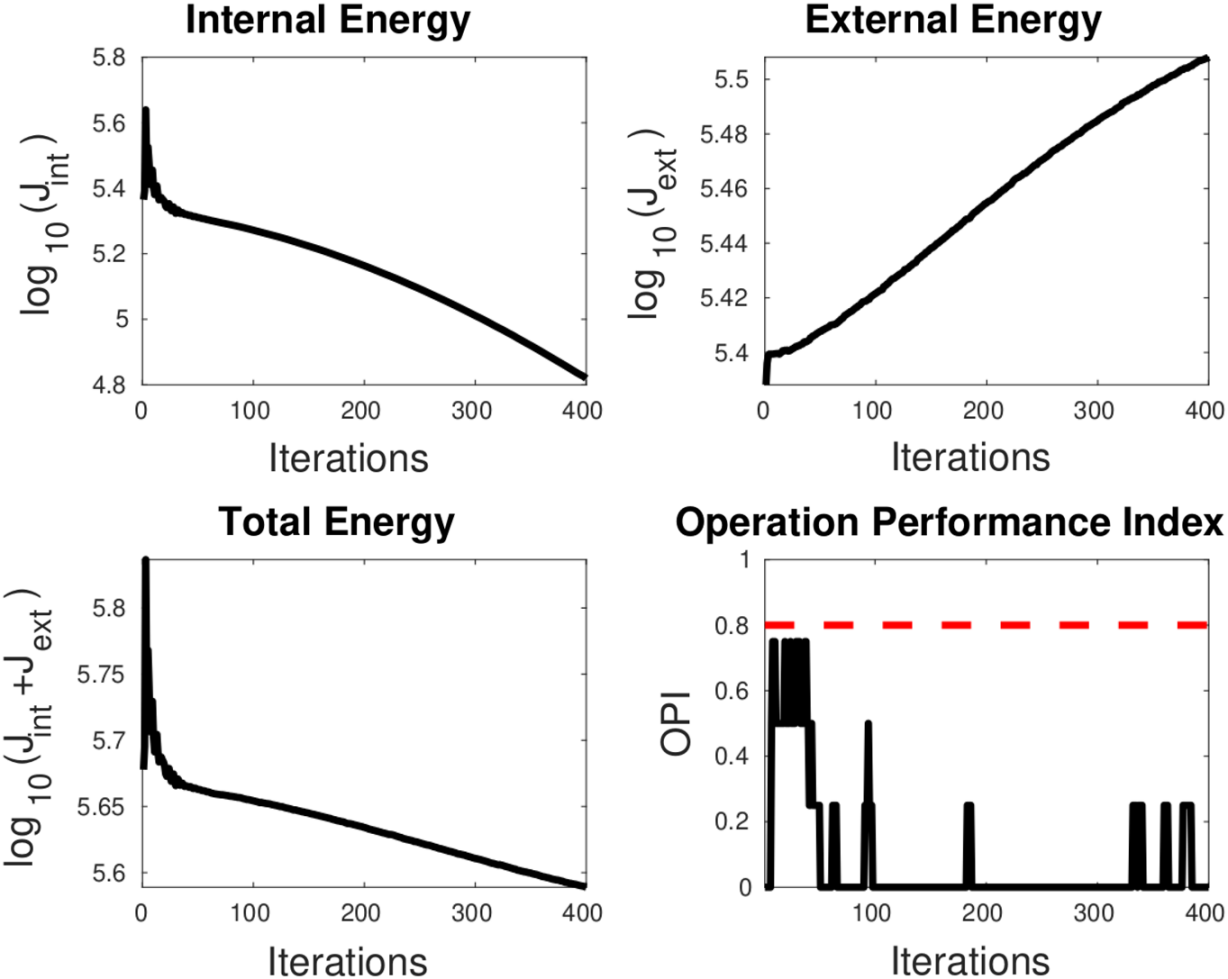
OPI trend (low value throughout) for LHS case of Fig. 7. The snake shrinks at all the steps and will ultimately shrink to a point. Dotted red line denotes the threshold.

**Figure 9:**
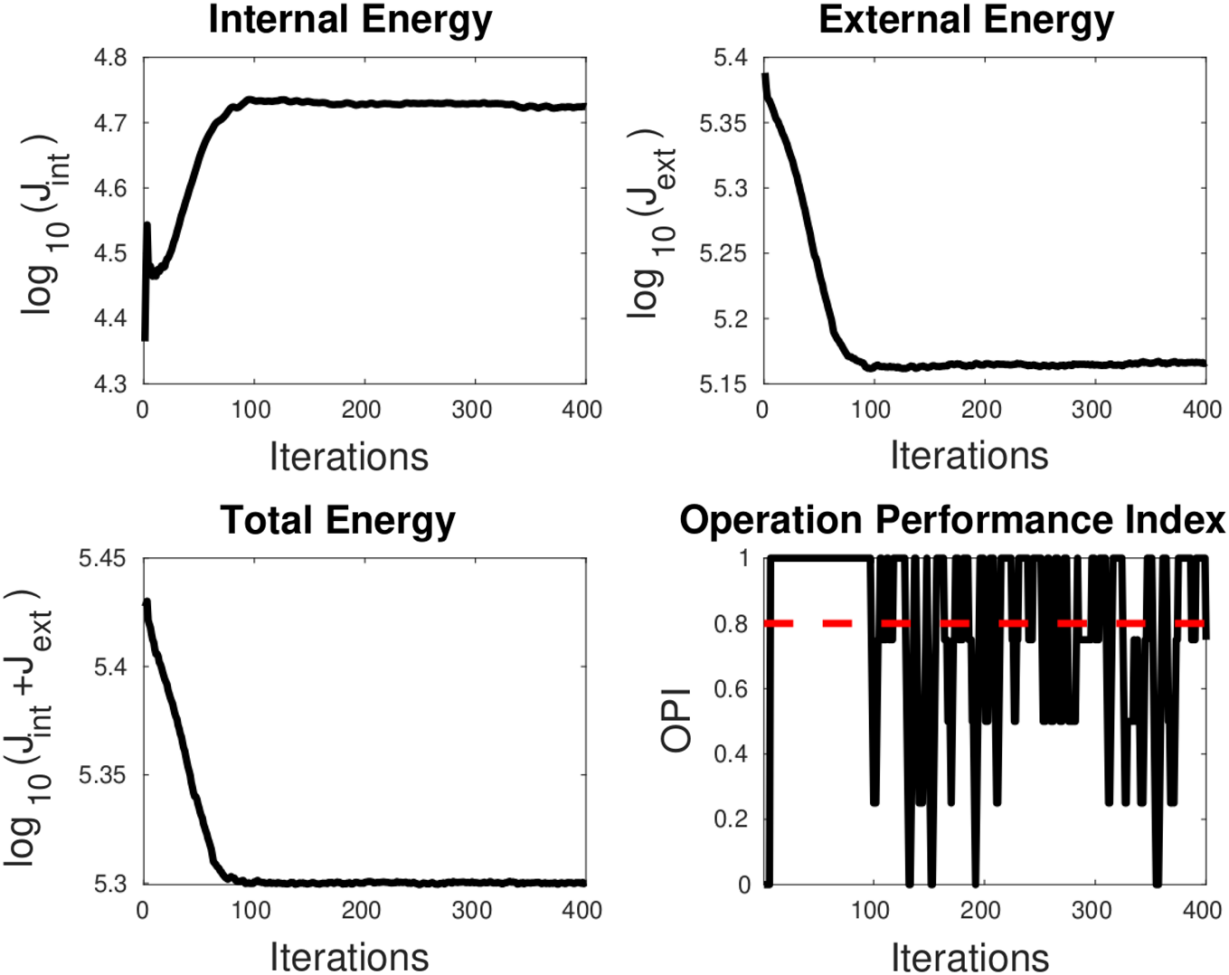
OPI trend (oscillating between 0 and 1) for RHS case of Fig.7. The snake gets stuck in a local minimum after about 100 iterations. Dotted red line denotes the threshold.

**Figure 10:**
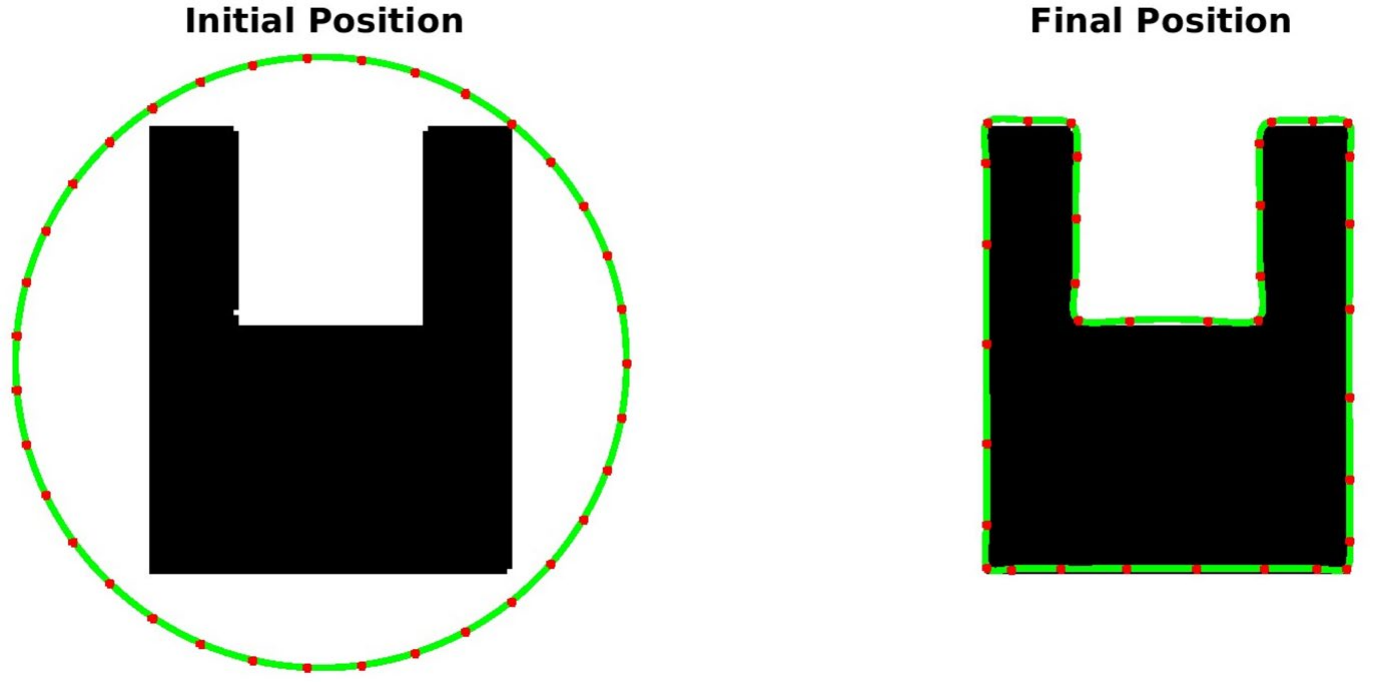
Performance of PICS with adaptive hyperparameters on a u-shaped cavity.

**Figure 11:**
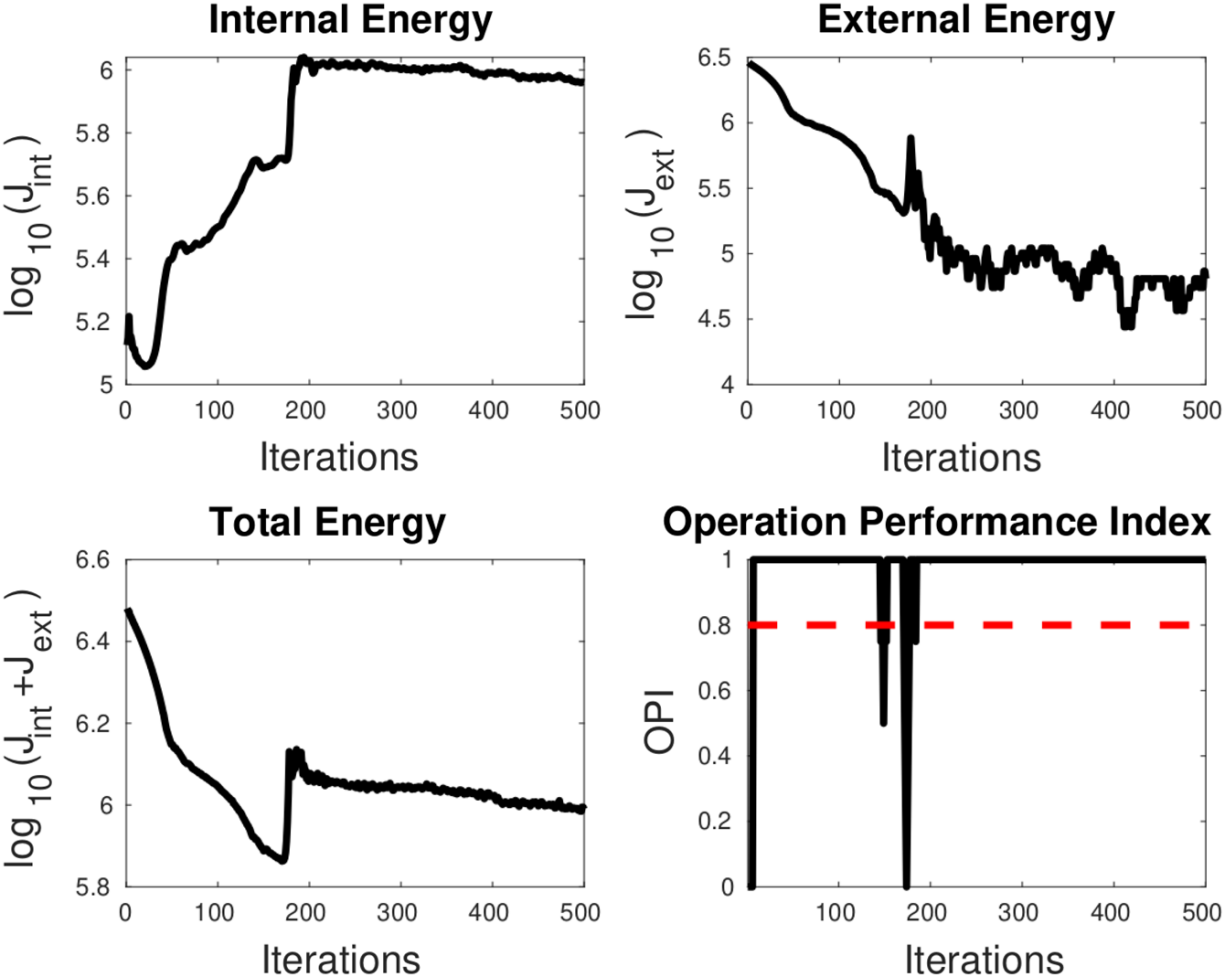
OPI trend of PICS with adaptive hyperparameters for cavity test case. Dotted red line denotes the threshold.

**Figure 12:**
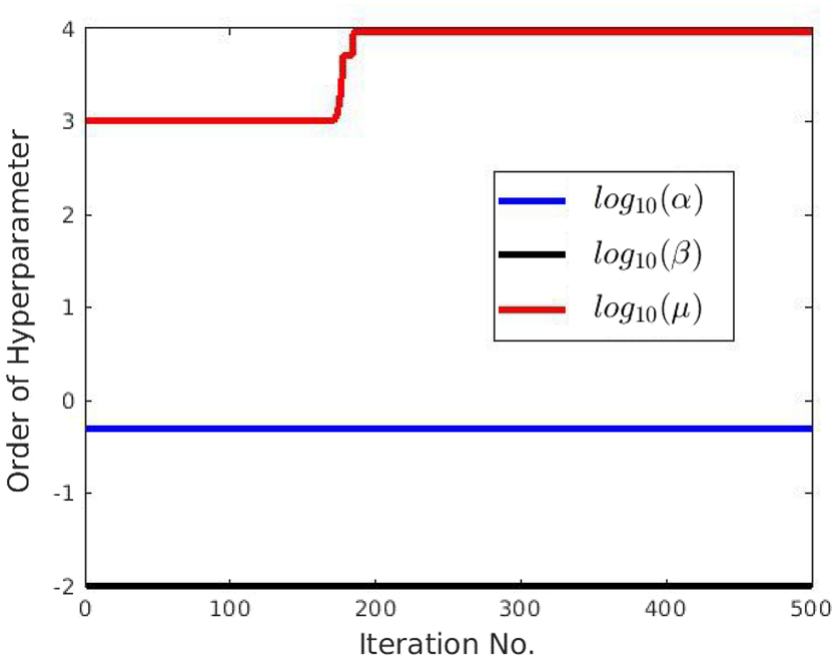
PICS hyperparameters tuning for cavity test case.

**Figure 13:**
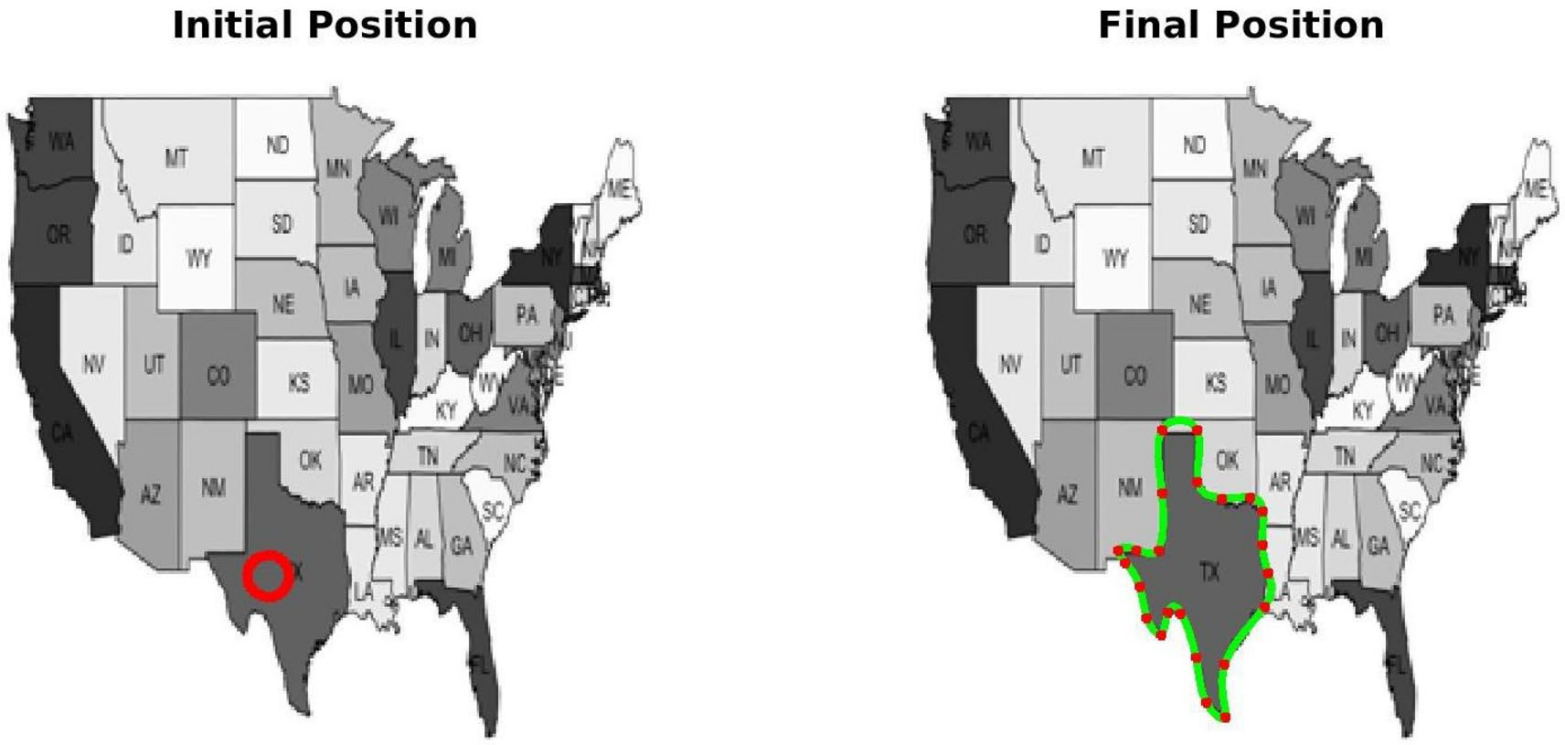
Segmentation of Texas state from the USA map. Left: Initial weights, Right: Optimized weights.

**Figure 14:**
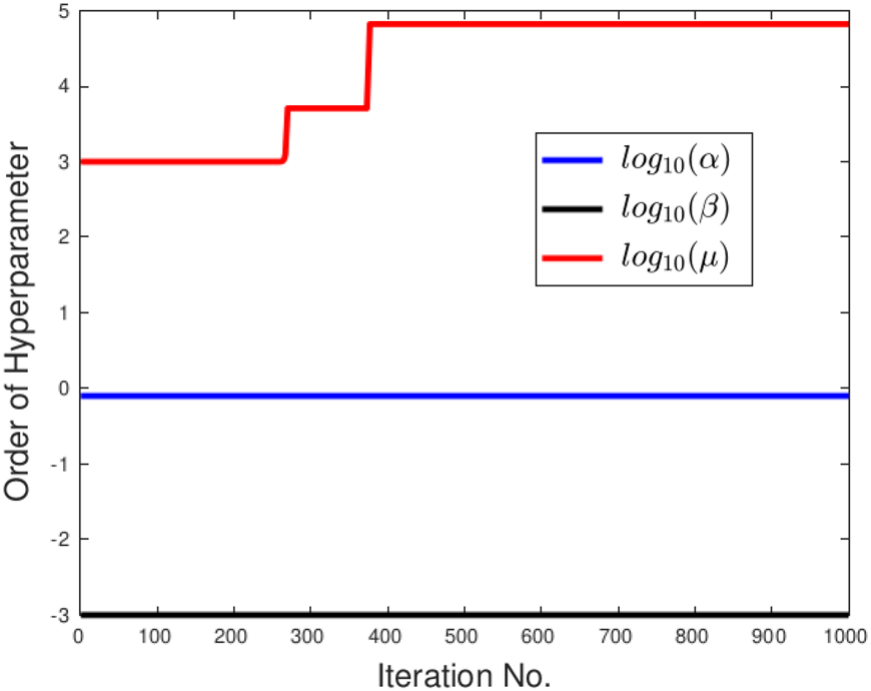
PICS hyperparameters tuning for the USA map case.

- **TC-3A and TC-3B: cavity case without adaptive hyperparameters.** Figure 7 displays two instances of bad minima. The first example shows a shrunken snake due to a high value of the bending coefficient. In contrast, the second shows the snake getting trapped in a local minimum because the increase in loss value caused by extension is greater than the drop in loss value due to the Chan–Vese loss. The loss history and OPI trend for each case are shown in Figs. 8 and 9 respectively. When examining the total loss history alone, it is difficult to determine whether the optimization is progressing correctly. However, looking at the OPI trend, we can see that its value is very low (zero) for the first case and fluctuates wildly between 0 and 1 for the second. Therefore, we can rely on the OPI to determine that PICS is stuck in a local minimum and requires adjustments to its hyperparameters to overcome this issue.
- **TC-3C: cavity case with adaptive hyperparameters.** Figure 10 demonstrates that PICS is capable of accurately capturing concave regions. Figures 11 and 12 provide additional information on this particular case, including the OPI score, loss history, and adaptive tuning of hyperparameters. These figures show that hyperparameters are adjusted as needed and a high value of OPI ensures that optimization is progressing in correct direction. No adjustments like gradient vector field^21^ or balloon forces^24^ are required in PICS framework.
- **TC-4: USA map with adaptive hyperparameters.** In the Figs. 13 and 14, we segment Texas state borders from the USA map. These figures show that hyperparameters are adjusted as needed. It should be noted that the PICS should be initialized near New Mexico region to provide large number of control knots for capturing sharp boundaries.

#### 3D case (TC-5)

**Figure 15:**
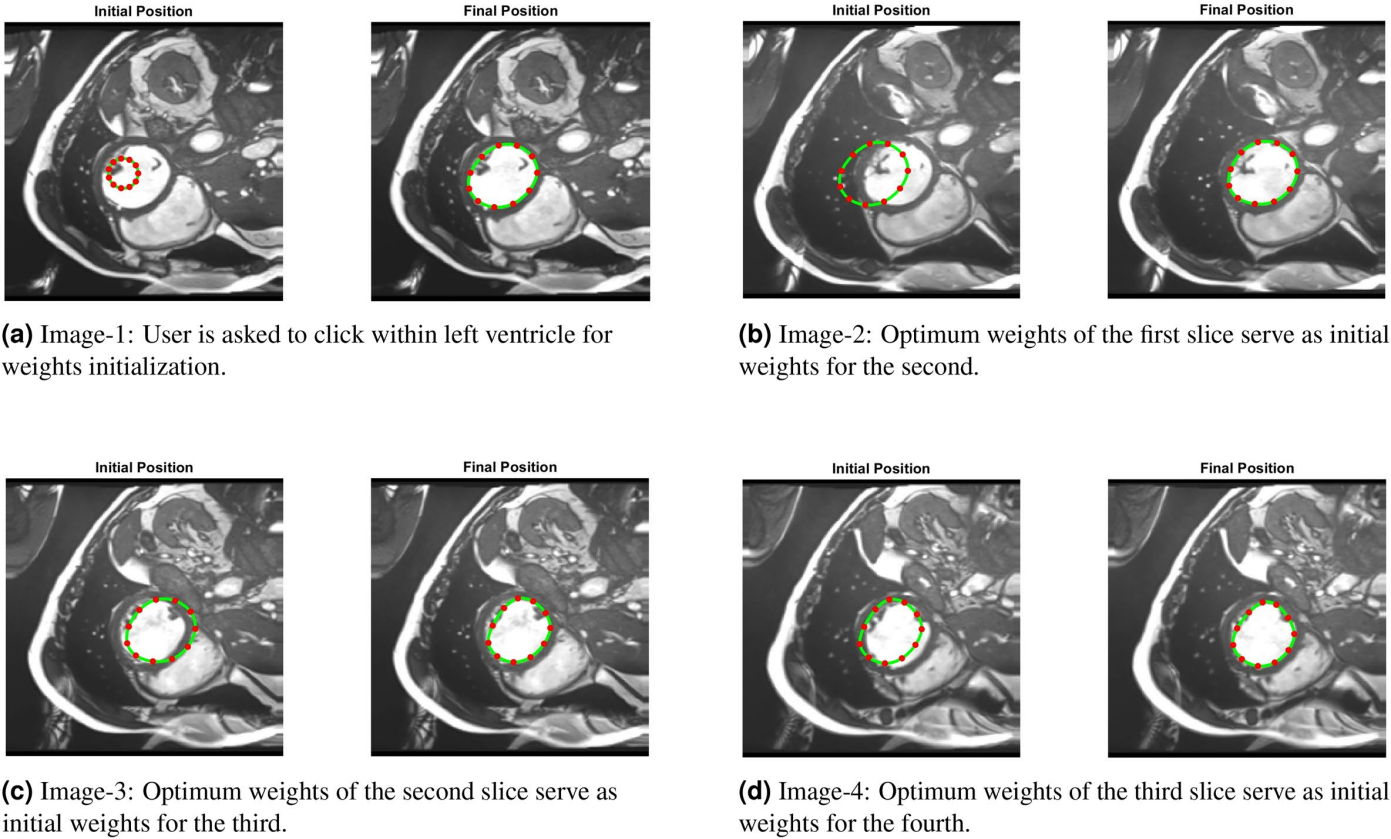
3D segmentation of left ventricle with PICS using transfer learning (for ED phase).

**Figure 16:**
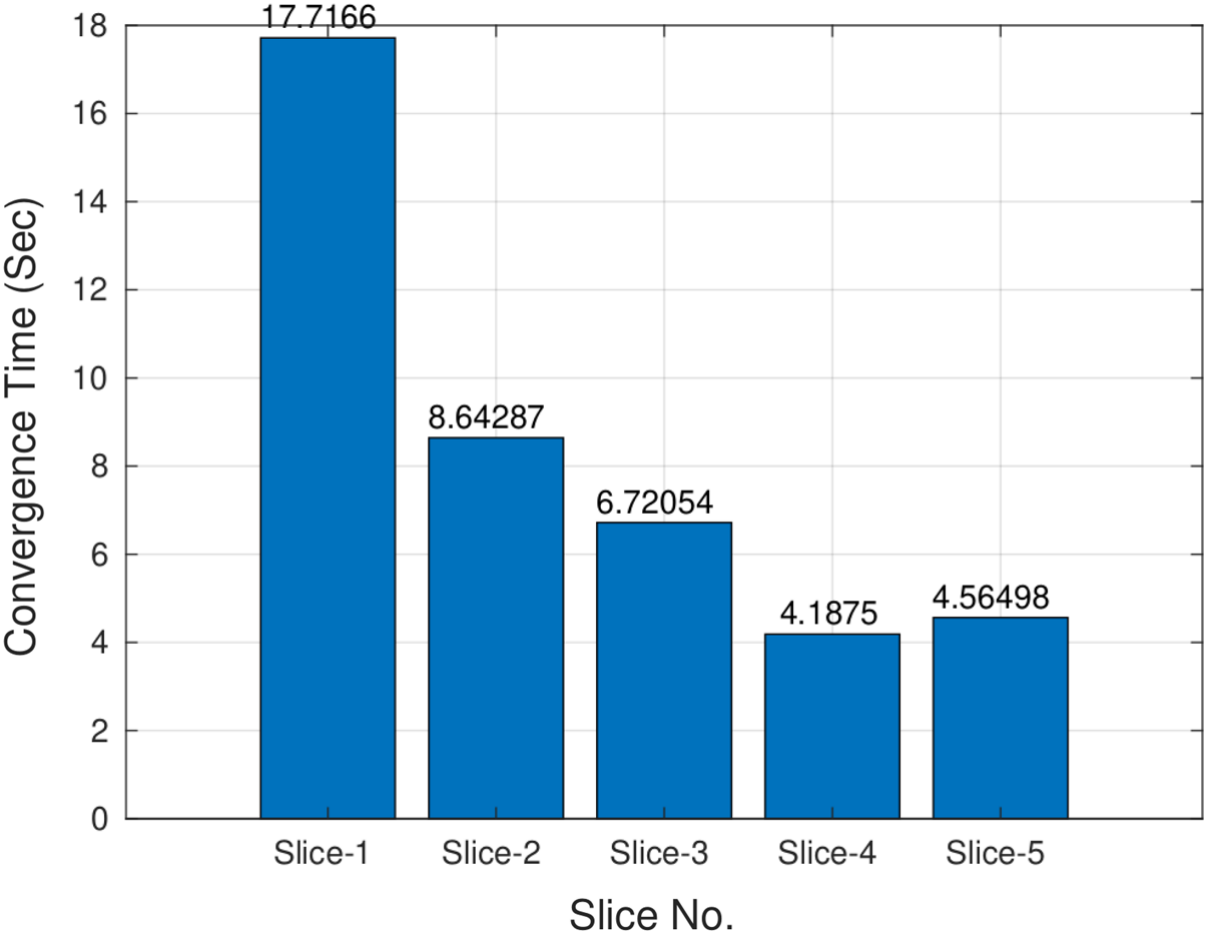
Faster convergence due to transfer learning.

**Figure 17:**
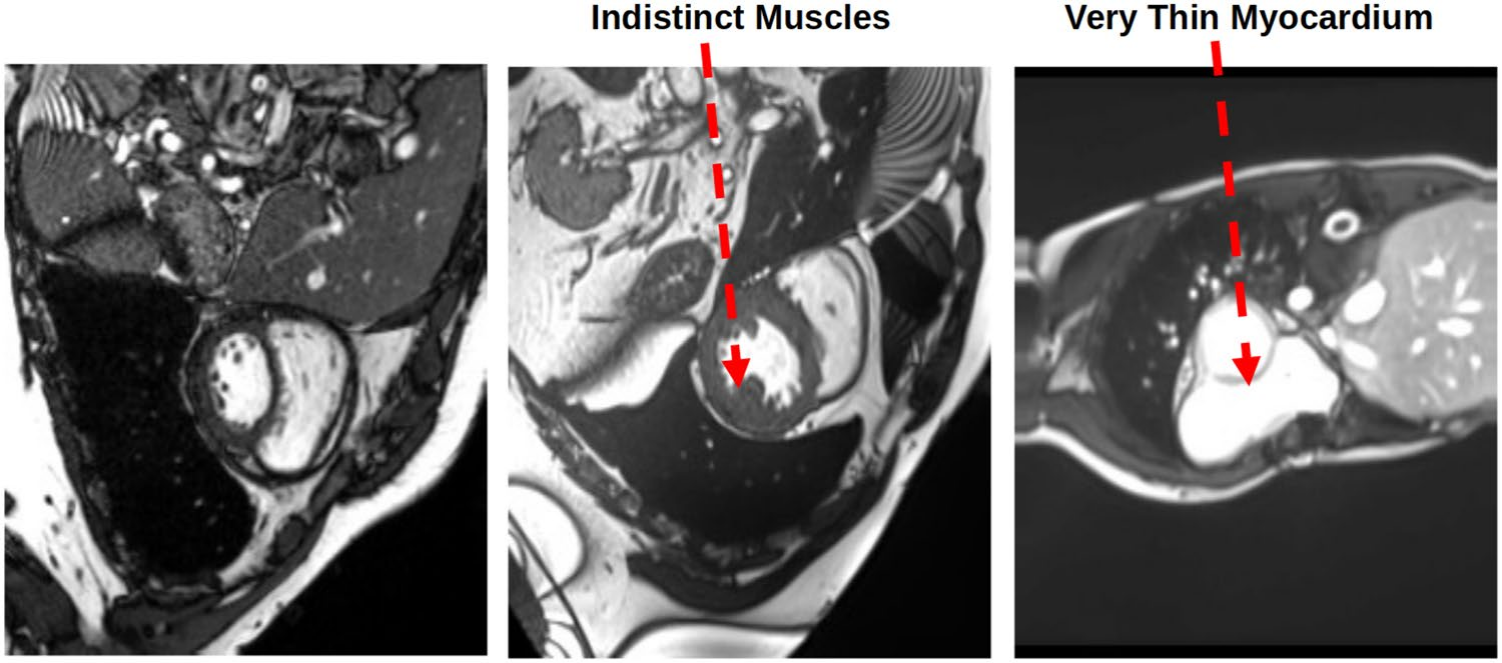
Hyperparameter selection for three distinct classes.

**Figure 18:**
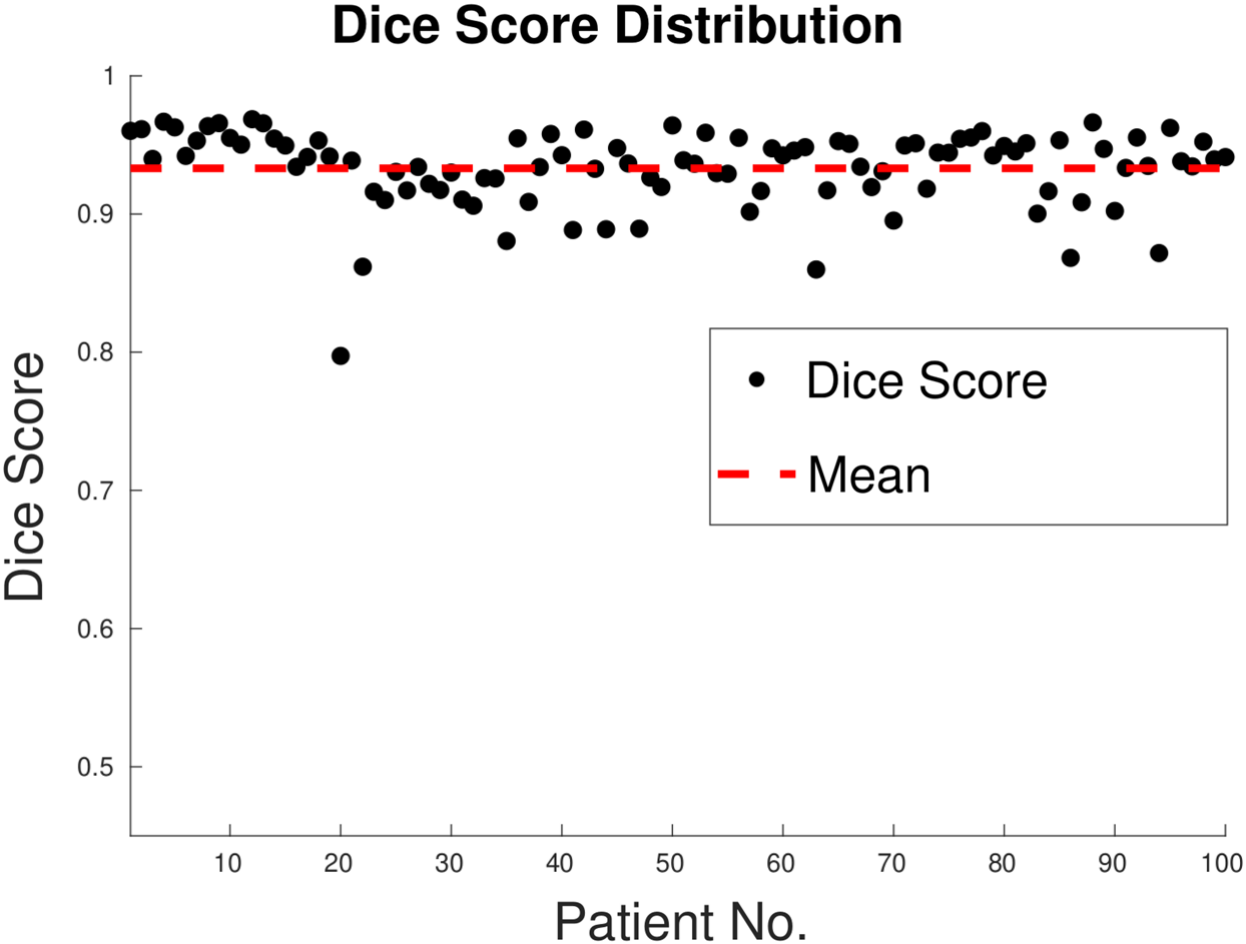
Performance of PICS in annotating the whole dataset consisting of 100 patients. Average dice score $= 0.933$.

- *3D PICS procedure*: Figure 15 demonstrates the 3D segmentation process of the left ventricle in the PICS framework. The initialization process begins with a single click within the left ventricle on the first MRI image. Subsequently, the optimal PIC weights obtained from the previous image are transferred as the initial condition for the next image. The transfer learning accelerates the convergence of the PIC to its optimal value. This iterative process is continued for all the remaining images.
- *Speed up*: Figure 16 illustrates an example of the speed-up in convergence due to this transfer learning process. Usually, the initial segmentation is finished in less than 30 s, while all the remaining segmentations also typically conclude within 30 s. Therefore, the full 3D segmentation takes about a minute to complete.
- *Hyperparameters selection*: The images in the ACDC dataset contain normal cases, indistinct muscle cases and cases with very thin myocardium (see Fig. 17). PICS uses different hyperparameter settings for different image categories in the ACDC dataset. For the normal case, the hyperparameters roughly have values of ($\alpha$,$\beta$,$\mu$,$\gamma$,$\sigma$)=(1e−1,1e−2,1e4,1e−5,1e7). For the indistinct muscles category, the value of $\sigma$ is increased by a factor of 10 while keeping all other hyperparameters fixed. Similarly, for the last class with very thin myocardium, the value of $\gamma$ is increased by a factor of 100-200 while keeping all other hyperparameters fixed. These hyperparameter settings are based on the observations from the ACDC dataset and have been found to provide good performance in their respective image categories.
- *Performance on ACDC dataset*: Figure 18 shows the performance of PICS on all the hundred patients’ data, with an average dice score of 0.933. The number of trainable parameters for all the cases is the same, which is 20 parameters.
- *Comparison with winners of ACDC challenge*: The best dice score(https://www.creatis.insa-lyon.fr/Challenge/acdc/results.html) is 0.96 while ours is a close 0.93. The first two winners of the challenge directly used the popular U-net^6^ architecture with dice loss or cross entropy as loss function. The third winner used M-Net^25^ architecture whose main difference with U-net resides in the feature maps of the decoding layers which are concatenated with those of the previous layer. The corresponding network was trained with a weighted cross-entropy loss.
  To summarize, PICS operates without the need for labels and utilizes only 20 parameters, in contrast to architectures like U-net that rely on millions of parameters, necessitating a substantial amount of high-quality data for effective training. It’s important to note that PICS doesn’t directly generate true masks; however, it can be utilized by experts to quickly produce initial masks. For example, in left ventricle segmentation, an expert can click once on the left ventricle, and PICS will provide a high-quality initial mask. The expert can then refine the positions of control knots (weights) and redraw a spline through them, reducing the labeling effort. Therefore, PICS demonstrates promise within the scope of our ongoing study, serving as an initial proof of concept.

## Discussion

In this paper, we introduced PICS—an interpretable, physics-informed algorithm for rapid image segmentation in the absence of labeled data. PICS is a novel algorithm that combines the traditional active contour model called snake with the physics-informed neural networks (PINNs). PICS inherits the unique qualities of its parent algorithms (snakes and PINNs), making it intuitive, mesh-free, and respecting the inherent physics of the traditional energy-based loss functions. The use of cubic splines over deep neural network as basis function and the treatment of spline control knots as design variables further increase its interpretability. We demonstrate that PICS is the first work to minimize the Chan–Vese loss in the snake framework and allows for easy integration of domain expertise via prior shape-based loss terms. PICS uniquely connects 3D segmentation with the solution of an unsteady PDE. By using this connection, as demonstrated by the outcomes on the ACDC dataset, PICS effectively utilizes transfer learning, leading to rapid and efficient segmentation.

**Figure 19:**
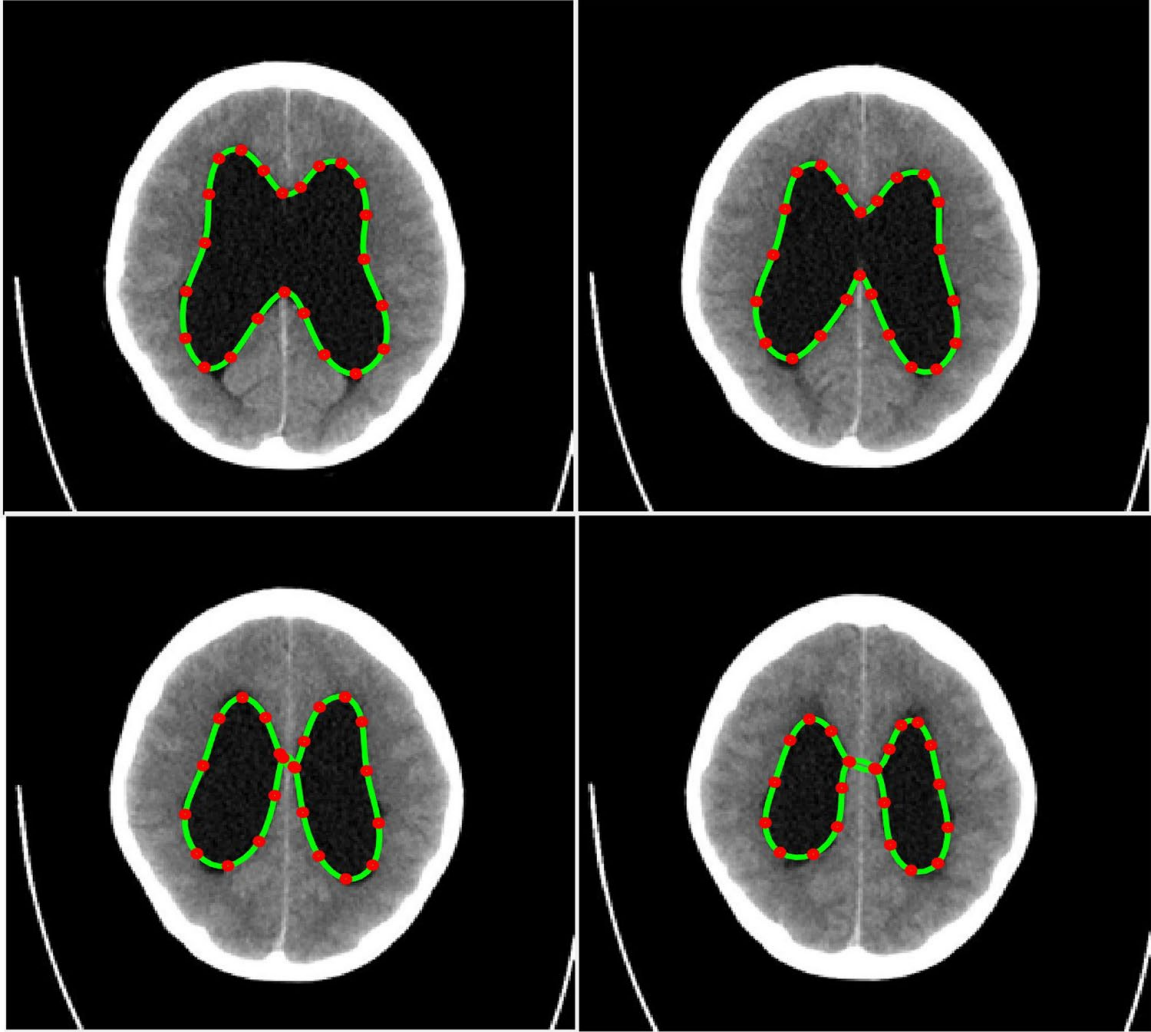
An example of topology change: 3D segmentation of enlarged ventricles of a hydrocephalus patient.

However, PICS also faces challenges in inverse parameter estimation and topology changes during 3D segmentation which are given below:

1. *Inverse parameter estimation.* It may be argued that because PINNs have been successful in both forward and inverse problems and PICS is derived from PINN, it should be possible to estimate hyperparameters by minimizing a loss function with hyperparameters treated as trainable weights. However, Zapf et al.^26^ explain that this may not be effective. Even when the predicted segmentation is correct, all three terms of the PICS loss function, including shape regularization, region-based loss, and shape-based loss, may not be equal to zero. Depending on the image complexity, the ratio of region-based loss to other losses may be the most trustworthy at times, while the ratio of shape-based loss to others may be most trustworthy at other times. Therefore, in this work, hyperparameters were chosen through trial and error. For simpler images, like those in Fig. 10, we can select the ratio of region-based loss to other losses as the most trustworthy and automate the hyperparameter selection process. Readers would appreciate that the proposed OPI effectively implements the suggestion by Zapf et al.^26^.
2. *Topology change.* The PICS framework for 3D segmentation has a limitation regarding images that change topology during segmentation. In such cases, multiple initializations are required, but the number of initializations needed is fixed based on the topology of the first image. This limitation is demonstrated in Fig. 19, where the 3D segmentation of CT scans of a hydrocephalus patient starts with one object in the first image but breaks into two parts in the fourth image, causing PICS to struggle with the increased number of parts. This issue may affect the accuracy and efficiency of the segmentation in cases where topology changes occur frequently.
3. *Less number of heart section images.* If there are only a few slices available for a specific cardiac cycle phase, such as end-diastole (ED), it could result in a significant change in the size of the left ventricle across adjacent slices, which can affect the accuracy of segmentation. Hence, having a larger number of slices available for a given phase is better for accurate segmentation of the left ventricle.
4. *Highly heterogeneous objects*: Chan–Vese loss focuses on homogeneity/uniformity within the contoured region due to which PICS may encounter challenges when dealing with highly heterogeneous objects, like tumor.

Overall, PICS shows great potential as a scientific machine learning approach, both for direct image segmentation and for aiding domain experts in generating data labels to train deep neural networks in the future.

### Supplementary Information

Supplementary Information.

## Data Availability

The details of the data used is as follows: (1) CT scan of enlarged ventricles of hydrocephalus patient (Case courtesy of Paul Simkin, Radiopaedia.org, rID: 30453). The source of data is: https://radiopaedia.org/cases/obstructive-hydrocephalus. (2) MRI scans of cardiac data of 100 patients from the ACDC dataset^13^ in the ED, i.e., End-Diastolic phase. Source:https://www.creatis.insa-lyon.fr/Challenge/acdc/index.html.
