# Supplementary material for: Physics informed contour selection for rapid image segmentation
Source: Sci Rep. 2024 Mar 24;14:6996. doi: 10.1038/s41598-024-57281-x (PMC10961308; doi:10.1038/s41598-024-57281-x)
Supplement: Supplementary file 1 — Supplementary Information. [file 41598_2024_57281_MOESM1_ESM.pdf]

Physics Informed Contour Selection for Rapid Image Segmentation (Supplemental Document)

This is supplementary document for the article titled "PICS in Pics: Physics Informed Contour Selection for Rapid Image Segmentation"

1. BRIEF REVIEW OF SNAKE AND PINN

Brief Review of Snake Model

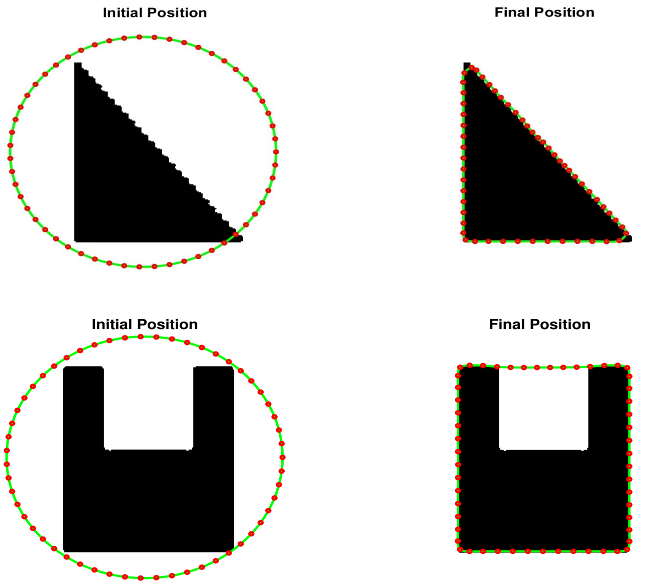

Fig. S1. Image segmentation with a naive image gradient-based snake. In the cavity case, please note that it gets stuck to a local minima.

Consider fig.S1 in which the snake (or deformable contour) has maximum energy at the start, and following the minimization process, it converges to the object's boundary. Mathematically, if the total energy of the snake is given by

$$J = \frac{\alpha}{2}\int_0^1 \psi_s^2 ds + \frac{\beta}{2}\int_0^1 \psi_{ss}^2 ds + J_{ext} \tag{S1}$$

where (s,t) denote space and time parameters respectively, ψ denotes the parametric spline used for segmentation contour, (α, β) are the coefficients, the sum of first two terms denotes the internal energy (J_{int}) of the snake and J_{ext} denotes external energy.

Then, the motion of the snake is governed by the following PDE:

$$\frac{\partial \vec{\psi}}{\partial t} = \alpha \frac{\partial^2 \vec{\psi}}{\partial s^2} + \beta \frac{\partial^4 \vec{\psi}}{\partial s^4} - \nabla J_{ext} \tag{S2}$$

The internal energy controls the smoothness of snake and it is independent of the data. However, J_{ext} is an image dependent, edge-based functional. For example, if I is the image, then a simple

gradient-based J_{ext} could be $J_{ext} = -\int_0^1 |\nabla I|^2 ds$. For such functionals, a differentiable function for the gradient of J_{ext} with respect to control knots cannot be found. However, if J_{ext} is a region-based functional, then the expression for ∇J_{ext} with respect to control knots cannot be directly found. If the object has weak gradients and the image is noisy, the denoising also removes the object boundary. In such cases, region-based loss functions are beneficial, but the traditional snake framework is not suitable for their implementation. For more details, please refer to [1–3].

Brief Review of PINNs

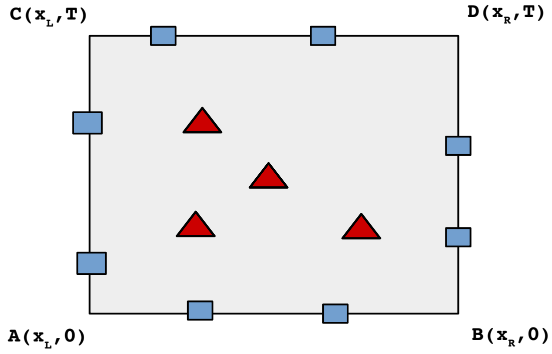

Fig. S2. Distribution of collocation (red triangles) and boundary points (blue rectangles) in the computational domain.

In a typical PINN, the solution of PDE is approximated by a deep neural network. The training data, which consists of collocation and boundary points (see fig.S2), are randomly distributed in the computational domain. For example, consider the following one-dimensional (1D) unsteady PDE.

$$\frac{\partial}{\partial t} u(x,t) + \mathcal{N} u(x,t) = R(x,t), (x,t) \in \omega \tag{S3}$$

$$u(x,t) = B(x,t), (x,t) \in \partial\omega, \tag{S4}$$

$$u(x,0) = F(x), x \in (x_L, x_R), \tag{S5}$$

where $\mathcal{N}$ is a nonlinear differential operator and $\partial\omega$ is the boundary of the computational domain ω. We approximate u with a n-layered deep neural network ψ such that

$$\psi = \psi(z; W_1, W_2 ... W_n, b_1, b_2, ... b_n) = W_n(...(\phi(W_2(\phi(W_1 z + b_1)) + b_2))...) + b_n \tag{S6}$$

where $z = [x,t]^T$denote sampling points, (W_i, b_i) denote model parameters and ϕ denotes nonlinearity. For PINNs, z are randomly selected, but after selection, they remain fixed. If we denote the errors in approximating the PDE, BCs, and IC by $\vec{\xi}_f$, $\vec{\xi}_{bc}$ and $\vec{\xi}_{ic}$ respectively. Then, the expressions for these errors are as follows:

$$\vec{\xi}_f = \frac{\partial \vec{\psi}}{\partial t} + \mathcal{N}\vec{\psi} - \vec{R},\ on\ (\vec{x}_f, \vec{y}_f) \tag{S7}$$

$$\vec{\xi}_{bc} = \vec{\psi} - \vec{B},\ (\vec{x}_{bc}, \vec{t}_{bc})_{side\ faces} \tag{S8}$$

$$\vec{\xi}_{ic} = \vec{\psi}(.,0) - \vec{F},\ (\vec{x}_{bc}, \vec{t}_{bc})_{bottom\ face} \tag{S9}$$

For shallow networks, $\frac{\partial \vec{\psi}}{\partial t}$ and $\mathcal{N}\vec{\psi}$ can be determined using hand calculations. However, for deep networks, we have to use finite difference methods or automatic differentiation [4]. The latter is preferred for its computational efficiency. We can recast the PDE, BC, IC system to an

optimization problem by minimizing an appropriate loss function. The loss function J to be minimized for a PINN is given by

$$J = \frac{\vec{\xi}_f^T \vec{\xi}_f}{2N_f} + \frac{\vec{\xi}_{bc}^T \vec{\xi}_{bc}}{2N_{bc}} + \frac{\vec{\xi}_{ic}^T \vec{\xi}_{ic}}{2N_{ic}}, \tag{S10}$$

where N_f, N_{bc}, and N_{ic} refer to the number of collocation points, boundary condition points in left and right faces, and initial condition points at the bottom face, respectively. We can see that we have chosen a least square loss function. Now, any gradient based optimization routine may be used to minimize J. For more details, please refer [5]

2. MATLAB CODES

Codes are available at: https://github.com/vikas-dwivedi-2022/Physics-Informed-Contour-Selection. This collection of MATLAB scripts offers an overview of the methodology detailed in the original paper. It's important to note that the PICS is still in its developmental stages, with factors like the number of control knots and initialization position yet to be thoroughly explored. Additionally, there's room for enhancement, particularly regarding inverse parameter estimation.

REFERENCES

1. M. Kass, A. Witkin, and D. Terzopoulos, "Snakes: Active contour models," Int. journal computer vision **1**, 321–331 (1988).
2. G. Sapiro, *Geometric partial differential equations and image analysis* (Cambridge university press, 2006).
3. R. Szeliski, *Computer vision: algorithms and applications* (Springer Nature, 2022).
4. A. G. Baydin, B. A. Pearlmutter, A. A. Radul, and J. M. Siskind, "Automatic differentiation in machine learning: a survey," J. Marchine Learn. Res. **18**, 1–43 (2018).
5. M. Raissi, P. Perdikaris, and G. E. Karniadakis, "Physics-informed neural networks: A deep learning framework for solving forward and inverse problems involving nonlinear partial differential equations," J. Comput. physics **378**, 686–707 (2019).
